# Causes of Caries

**Published:** 1856-06

**Authors:** J. Taft


### CAUSES OF CARIES.

CONTINUED FROM OCT. NUMBER.

BY J. TAFT.

The causes of caries of the teeth may be considered under two general divisions. *Predisposing* and *exciting*. Of the predisposing causes, some are original, others accidental.

The original development of the constitution may be defective, this may result from original or accidental defect of the parent; but more certainly from the former. Constitutional characteristics are transmissable, and a *defect* is as certainly transmissible as any thing else. The fetus during gestation, may have formed germs from which perfect organs can never be developed, they may be more or less defective according to the condition of the mother. She may also be subject to accidental conditions that will seriously affect the fetus.

The child after birth is exposed to injurious impressions that will to a greater or less degree render the development defective. Imperfect nourishment, the diseases and various functional derangements to which childhood is subject, are some of the circumstances that modify the perfect development of the teeth.

A diseased condition, or functional derangement will interrupt the proper elimination, and perfect upbuilding of the materials necessary for the perfect structure. Or even any thing that will destroy the equilibrium of action in the system may be detrimental to the teeth.

The teeth will sometimes exhibit the peculiarities of one parent and at other times the other; and sometimes an intermediate character. When the organic imprint of a parent is enstamped upon the teeth, we find exactly the same class of teeth decay, and at the same point upon the teeth, and at about the same age.

In such cases an hereditary defect is manifest, it cannot be accidental; the fact of its occurring upon the same class of teeth at the same point, and at about the same period of life, precludes any such conclusion. Hereditary taint then, we regard as a predisposing cause of caries. Impaired vitality is a predisposing cause of caries.

Not only is diminished vitality in the teeth or their contiguous parts a cause of decay, but the impairment of the vitality of the general system has the same tendency. The vital vigor of the teeth depends upon that of the general system, and when there is no local cause acting perniciously, corresponds with it; so that when the general system is in the most perfect condition, the teeth possess the greatest power of resistance to injurious agents.

This resisting power is comparatively feeble; but this is compensated for to some extent, by the peculiar structure of the teeth, and the materials of which they are formed, being naturally less liable to decomposition than any other part of the human body. Nevertheless, their welfare depends much upon the retention of the healthy vitality, and this upon that of the general system. A dead tooth surrounded by similar circumstances will decay far more rapidly than a living one, and hence the conclusion that vitality resists injurious agents, and that resistance will correspond with that vitality.

All febrile conditions facilitate decay, and frequently in two ways, by diminishing the general vitality, and by changing the secretions of the mouth, so that they act injuriously upon the teeth. There is generally accompanying such conditions, inflammation of the dentine. Always when there is a general inflammatory tendency, the dentine partakes of that condition, and is then quite susceptible to injury.

All diseases that impair the vitality and change the secretions, may be considered predisposing causes of decay. Some diseases accomplish more than this, dyspepsia not only accomplishes all this bnt does vastly more, it prepares in the stomach an acid which is thrown almost constantly upon the teeth, and acts upon them with great energy.

Residence in miasmatic regions predisposes to caries of the teeth, by inducing unfavorable conditions. Diminished vitality may result either from constitutional or local causes.

The local causes are such as produce an irritable or diseased condition of the immediate parts, or an abnormal condition of the dentine, without the power to effect its decomposition. Local causes producing diminished vitality are not so formidable in their nature, and are far more easily controlled than constitutional.

Many medicinal agents are regarded as predisposing causes of caries. Among these mercurials occupy a prominent place. This is accomplished by vitalizing the secretions of the mouth, producing an abnormal condition of the periosteum about the fangs of the teeth, the mucous follicles and salivary glands. These agents may act injuriously, by impairing the vigor of the constitution.

The opinion is entertained by some, that the abnormal action of the absorbents, induced by mercurials predisposed the teeth to decay.

Operations performed upon the teeth at an improper time, and in an improper manner, predispose them to decay. Their vitality may thus be impaired or a diseased condition established, or the part operated upon permitted to remain rough, so that foreign substance will be retained, become vitiated, and act upon it.

Often by the improper use of the file, extensive inflammation of the dentine supervenes, and the death of the tooth sometimes follows, and the contiguous parts become involved in disease. Artificial substitutes imperfectly adapted, are often the occasion of caries, not that clasps or the edges of the plate act directly on the tooth to destroy it, but agents arise upon their presence that do act upon the teeth, and particularly when the material used is not of the right quality.

A want of proper exercise in mastication, induces a condition that facilitates decay. It prepares agents to act upon the teeth rather than communicate any predisposition to the teeth themselves. Substances of all kinds are deposited with far greater rapidity when the teeth are idle.

The teeth cannot with impunity undergo rapid transitions from one extreme of temperature to another, or even such extremes as may be endured by the surrounding parts. By it inflammation of the dentine may be set up, their vitality diminished, and even in cases of friable teeth, checking of the enamel will occur, and in this way a condition arise that will facilitate decay.

(TO BE CONTINUED.)

